# Accurate Kidney Pathological Image Classification Method Based on Deep Learning and Multi-Modal Fusion Method with Application to Membranous Nephropathy

**DOI:** 10.3390/life13020399

**Published:** 2023-01-31

**Authors:** Fang Hao, Xueyu Liu, Ming Li, Weixia Han

**Affiliations:** 1 College of Data Science, Taiyuan University of Technology, Taiyuan 030024, China; 2 Department of Pathology, Second Hospital of Shanxi Medical University, Taiyuan 030001, China

**Keywords:** membranous nephropathy, kidney pathology, deep learning, multi-modal fusion, whole-slide images, immunofluorescence images

## Abstract

Membranous nephropathy is one of the most prevalent conditions responsible for nephrotic syndrome in adults. It is clinically nonspecific and mainly diagnosed by kidney biopsy pathology, with three prevalent techniques: light microscopy, electron microscopy, and immunofluorescence microscopy. Manual observation of glomeruli one by one under the microscope is very time-consuming, and there are certain differences in the observation results between physicians. This study makes use of whole-slide images scanned by a light microscope as well as immunofluorescence images to classify patients with membranous nephropathy. The framework mainly includes a glomerular segmentation module, a confidence coefficient extraction module, and a multi-modal fusion module. This framework first identifies and segments the glomerulus from whole-slide images and immunofluorescence images, and then a glomerular classifier is trained to extract the features of each glomerulus. The results are then combined to produce the final diagnosis. The results of the experiments show that the F1-score of image classification results obtained by combining two kinds of features, which can reach 97.32%, is higher than those obtained by using only light-microscopy-observed images or immunofluorescent images, which reach 92.76% and 93.20%, respectively. Experiments demonstrate that considering both WSIs and immunofluorescence images is effective in improving the diagnosis of membranous nephropathy.

## 1. Introduction

Membranous nephropathy (MN) is also known as membranous glomerulonephritis. Membranous nephropathy (MN) is one of the most prevalent conditions responsible for nephrotic syndrome in adults. The incidence of nephrotic syndrome in the elderly population in China is 23.4% [1]. MN is a glomerular disease with an increasing incidence. Its clinical manifestations include proteinuria, edema, and hypoalbuminemia. Hypertension and high cholesterol also frequently occur. However, these clinical manifestations are nonspecific, and membranous nephropathy must be diagnosed by renal biopsy. It is pathologically characterized by diffuse glomerular basement membrane (GBM) thickening and subepithelial immune complex deposition.

**Figure 1:**
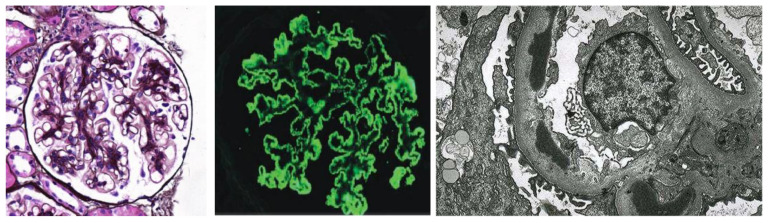
The glomeruli were observed by the three main techniques of Light microscopy $\left(\mathrm{PASM}\times 400\right)$, Fluorescence microscopy $\left(\times 400\right)$, and Electron microscopy $\left(\times 5000\right)$ used in the pathological examination of the renal biopsy.

Renal pathology plays a crucial role in the clinical diagnosis, management, and prognostic evaluation of MN. Combining at least two or more of the three types of microscopy—light microscopy (LM), transmission electron microscopy (TEM), and fluorescence microscopy—is required for accurate renal pathology diagnosis. The glomeruli observed by three microscopes are shown in Figure 1. The light source system of the optical microscope is a common light source, which can magnify the tissue section being observed by 400 times. Tissue morphology at the cellular level can usually be observed under a light microscope. The relative light mirror of the transmission electron microscope has a magnification that can reach 8000 times. It plays an important role in the early diagnosis of membranous nephropathy. Compared with LM and fluorescence microscopy, TEM is expensive and demanding in the environment. Therefore, LM and fluorescence microscopy are more common, and the images observed by them are easier to obtain. Despite these advanced techniques, observation of pathological images is performed by experienced senior pathologists. They need to manually observe glomeruli one by one, a process that is subjective and laborious. Automated and accurate methods to provide physicians with diagnostic aids are needed and promising.

Because kidney pathological images are more complex and the tissues or cells to be evaluated on medical images are so similar, it is difficult for non-specialists to differentiate between them. Therefore, all of the medical image analysis and processing methods proposed in the previous ten years are non-generalized or model-based, meaning that when medical data change, researchers must devote a great deal of time and effort to designing a new method. These problems can be easily solved due to the advantages of deep learning, such as robustness and simplicity. In 2013, Ciresan first used deep learning for the detection of mitosis in pathological slices, as described in the document [2], which proved that deep learning is helpful for the research and analysis of pathological images. In 2016, researchers analyzed lung cancer samples through artificial intelligence technology [3], which can provide indicators for patient prognosis analysis. Since then, artificial intelligence techniques have been extensively explored and implemented in the field of intelligent pathological diagnosis [4]. Thus far, AI-assisted diagnosis has done a good job of finding cancer in lung, liver, breast, and pancreas pathological slice images.

Deep convolutional neural networks (CNNs) are commonly utilized for the supplementary diagnosis of WSIs as a result of their successful application in the kidney pathology area. The tissue structure of kidney disease images is complex, and the identification of lesion features is easily affected by factors such as staining and background tissue, which make the identification results inaccurate. Therefore, studying the detection and recognition methods of pathological images and assisting the relevant hospital staff in performing qualitative and quantitative analysis have become current and future research hotspots. Gurcan et al. [5] trained a CNN on kidney pathological WSIs to identify glomeruli with an accuracy of 87.8 percent. Pedraza et al. [6] implemented the classification of glomerular and non-glomerular areas in kidney disease images using deep neural networks in 2017. Barros et al. [7] created a pathological computing system to detect lesions in digital kidney pathological images to detect proliferative glomerular lesions with an 88.3% accuracy rate. Gallego et al. [8] implemented glomerular classification and WSI detection using overlapping sliding windows based on the AlexNet framework. They confirmed the need for color normalization to identify glomeruli with varied degrees of staining. Marsh et al. [9] developed a CNN model to recognize and categorize sclerotic and non-sclerotic glomeruli in WSIs of frozen section kidney cases. By expanding a pre-trained network model, a diagnostic accuracy similar to that of experienced renal pathologists was achieved with only 48 abnormal sections. Kannan et al. [10] developed a deep learning framework for glomerular identification and segmentation in three kinds of stained images of kidney pathology specimens from 171 chronic kidney disease patients with an accuracy of up to 82.02%. Ginley et al. [11] built a CNN model to detect glomerular boundaries and nuclei from WSIs and defined a set of features to quantify and classify diabetic nephropathy. Uchino et al. [12] used 283 pathological images of kidney biopsies and a fine-tuned InceptionV3 convolutional neural network to classify 7 types of glomerular lesions, and the accuracy rate of glomerular global sclerosis classification was as high as 98.6%. Chagas et al. [13] proposed an architecture that utilizes CNN to extract features and support vector machines for classification, achieving near-perfect average results on a binary dataset of proliferating and normal glomeruli. The algorithm proposed by Salvi et al. [14] was used to assess glomerulosclerosis by employing a fully automated approach to detect key tissues, such as glomeruli.

The primary method for applying deep learning to immunofluorescence images is to stack networks hierarchically. These works are able to recognize texture features. Only one literary work achieves this objective. Through the use of a convolutional neural network, Ligabue et al. [15] were able to identify the distribution and intensity of the glomerular deposit.

Here, the most pressing challenges in renal pathology’s image-based MN classification are highlighted. First, when pathologists make a clinical diagnosis, they often need to confirm the results of a variety of examination methods, including immunofluorescence, electron microscopy, and light microscopy. Moreover, only observing one of the microscope images from a light microscope or fluorescence microscope may not necessarily show the typical features of membranous nephropathy. Using only one modality, such as light microscopy, different glomeruli may still show features of different stages of the lesion, which has a confounding effect on the classification of MN. It is also a concern to improve the classification effect of membranous nephropathy under the premise of reducing the workload of labeling. Third, a whole-slide image (WSI) is a high-resolution image, and a kidney pathological WSI is generally gigabyte-level, which is a difficult problem for the training and testing of deep learning networks. To make sure the data are accurate and available, the lesion labeling work can only be performed by an experienced pathologist. This makes the labeling job more difficult, so it is difficult to obtain a lot of professionally labeled pathological images.

To address these issues, it is proposed to classify MN using a network framework that combines periodic acid-silver methenamine (PASM)-stained light-microscope images and IgG antibody-positive immunofluorescence images. This network framework is mainly divided into three stages. The first stage is the segmentation network, which separates the glomeruli from the original image. The second stage separately classifies glomeruli on images from the two modalities and obtains confidence coefficients. The third stage fuses the two confidence coefficients. This classification network framework segments glomeruli first, then fuses the classification results of two types of images: optical microscope images and immunofluorescence images, which can effectively improve the accuracy of MN classification.

The following is the outline for this paper: classification networks and multi-modal fusion methods are discussed in Section 2. The proposed classification algorithm is introduced in three stages in Section 3. Section 4 contains a description of the datasets and experiments used in this paper. Finally, Section 5 provides some conclusions.

## 2. Related Work

### 2.1. Classification Networks

As an important method of deep learning, deep convolutional neural networks are widely used in computer vision tasks, especially in image classification tasks, which use convolution to automatically extract features and determine image categories. AlexNet, as the earliest deep convolutional neural network, has attracted a lot of attention since it won the ImageNet competition classification task champion in 2012. Since then, classification algorithms based on deep convolutional neural networks have emerged one after another. At present, the most classic classification algorithms are VGGNet, GoogLeNet, Inception, ResNet [16], etc. CNN is quickly becoming a common technique for image classification screening, and its medical applications are vast. Using CNN to automatically learn discriminative features to categorize mammography lesions, Arevalo et al. [17] established a paradigm for feature learning for breast cancer diagnosis using CNN to automatically learn discriminative features. CNN is also commonly used to classify objects or lesions. Kawahara et al. classified skin lesions using a CNN with multiple procedures for processing images at varying resolutions. Jiao et al. used CNN to extract deep features at many levels, which improved the classification accuracy of breast cancer [18]. CNN is also used in bone age assessment and sex determination [19], tuberculosis detection [20], medical image alignment [21], medical image retrieval [22], and diabetic retinopathy detection [23]. However, these classification CNN algorithms also have certain limitations. They pay more attention to the overall information of the image, and the image details are eliminated in the process of convolution. The structure of renal pathological images is more complex, and the observation process pays more attention to the details of some specific tissues. The similarity of tissues makes the classification results of the CNN network easily disturbed. Therefore, it does not show high accuracy on a single type of renal pathology image.

### 2.2. Multi-Modal Fusion Methods

Multi-modal fusion is also called multi-source information fusion, and multi-sensor fusion. Multi-modal fusion refers to the process of combining the information of two or more modalities for prediction. During prediction, the effective information contained in a single modality is limited, so the results cannot be accurately predicted. The multi-modal fusion process excavates potential knowledge and realizes information supplementation by synthesizing the information of multiple modalities. Multi-modal fusion broadens the coverage of the information contained in the input data, improves the accuracy of the prediction results, and obtains a robust prediction model.

Multi-modal fusion is used in applications such as audiovisual speech recognition (AVSR) [24], multi-modal emotion recognition [25,26], and medical-aided diagnosis [27].

Generally, fusion methods can be put into four groups based on the stage of fusion: signal-level fusion, data-level fusion, feature-level fusion [28], and decision-level fusion [29].

Pixel-level fusion is the most basic form of multi-modal image fusion. The image obtained after pixel-level image fusion has more detailed information, such as edges and textures. However, pixel-level fusion has certain limitations. Pixel-level fusion means higher computation and more computation time, and the resolution of the fused images should not be too different. Feature-level image fusion needs to first extract the feature information in the image and then process, analyze, and integrate this feature information to finally obtain the fused image features. The step of extracting key features results in improved computational speed and accuracy. Decision-level image fusion is to fuse the decision results of each modal image according to the actual problem. This method requires the least amount of computation, but its accuracy is highly dependent on the credibility of the decision-making process. At present, the area undergoing the most research and application is pixel-level image fusion. In addition, there is no general fusion algorithm, and a reasonable fusion model needs to be established according to the actual application scenario.

## 3. Methods

**Figure 2:**
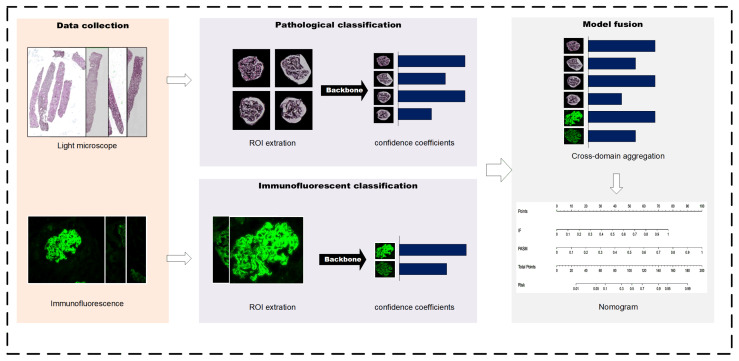
The workflow of this study.

This section demonstrates how to use PASM-stained whole-slide images and IgG immunofluorescence images to determine whether or not a patient has MN. Our suggested model’s whole design consists of three components. The process of our investigation is depicted in Figure 2. The framework consists of three modules: the module for the region-of-interest extraction, the module for confidence coefficient creation, and the module for cross-domain aggregation. First, the glomeruli are identified from the immunofluorescence pictures, or WSIs, by using the author’s previously trained networks, which exactly match the region-of-interest extraction module. The confidence-coefficient-generating module then combines many attention mechanisms to classify MN and derive the confidence coefficient for two multi-modal images. After the training phase of classification is complete, the fusion module combines the results of classification to produce a single label.

### 3.1. Region-of-Interest Extraction Module

According to the pathogenesis of membranous nephropathy (MN), MN is formed due to the deposition of immune compounds on the GBM of the glomerulus. Therefore, the diagnosis process for MN mainly focuses on the glomerulus. There are other tissues and noises, such as proximal and distal tubules, around the glomerulus that can alter the classification accuracy of the CNN, regardless of whether you are seeing WSIs or immunofluorescence images. It is necessary to preprocess the WSIs or immunofluorescence images, crop out the glomeruli, and suppress noise interference to accurately classify the glomeruli.

**Figure 3:**
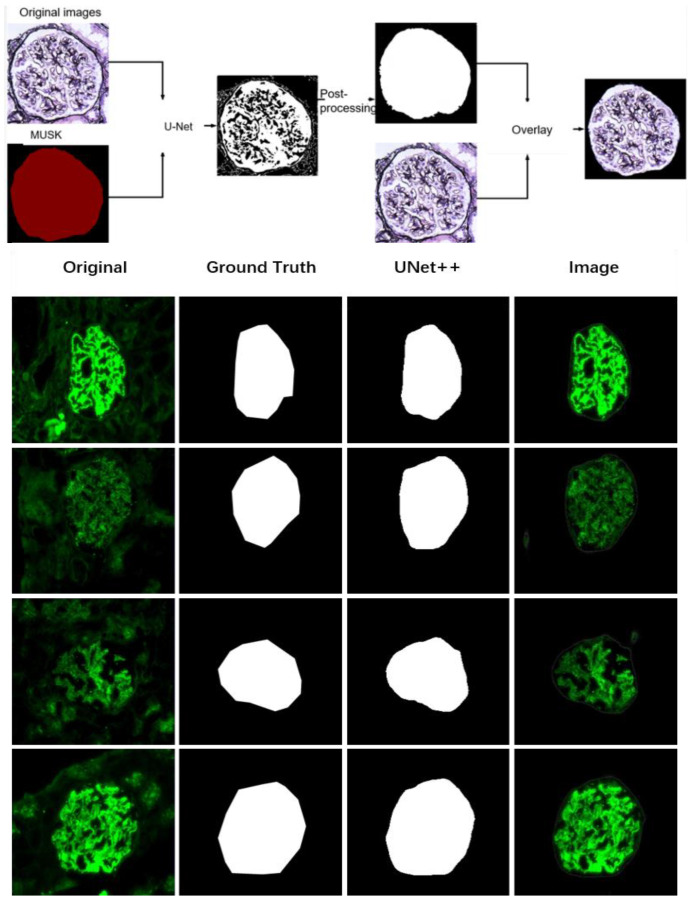
Glomerular segmentation on PASM staining $\left(\times 400\right)$ (**above**) WSIs and immunofluorescence images $\left(\times 400\right)$ (**below**).

Thus, the first step is to segment the glomeruli in WSIs and immunofluorescence images. This is performed with an automatic segmentation architecture based on deep learning algorithms. Segmentation results for glomeruli are shown in Figure 3.

The glomerular object locator (GOL) proposed in our previous work was used to efficiently localize and segment glomeruli in WSI or immunofluorescence images. Specifically, for the high-resolution and fine-grained characteristics of WSIs of pathological slides, our recently proposed GCPANet32 structure can segment each glomerulus from a kidney pathological WSI.

Glomeruli on immunofluorescence images are a salient object. UNet++ is utilized to pre-segment glomeruli on immunofluorescence images due to its superior ability to segment salient objects.

### 3.2. Confidence Coefficient Generation Module

In the clinical diagnosis of MN, the three inspection techniques of electron microscopy, immunofluorescence microscopy, and light microscopy are required to confirm each other, and generally, at least two inspection techniques are required. In our study, annotations for each image are derived from patients’ electronic pathology records, which are given by senior pathologists. Due to the diffuse character of MN, almost all glomeruli in the case of MN show immune complex deposition. Thus, all of the segmented images of glomeruli from the same recording are marked as “positive”, while those without MN are marked as “negative”.

Then, using PASM-stained WSIs and immunofluorescence images, a deep convolutional-neural-network-based classification model is made that could classify whether each glomerulus image is positive or negative on PASM-stained or immunofluorescence images. In the traditional CNN binary classification model, each image outputs a confidence coefficient in the last layer and then passes a threshold (usually 0.5) to determine whether it is in a positive or negative category. However, different thresholds calculate different recall and precision. A fixed threshold cannot fit every model. As a result, the image category, which is the output of this phase, is replaced with the positive confidence coefficients of $C_{P_{i}}$ and $C_{F_{i}}$ of the PASM-stained WSIs and immunofluorescence images to eliminate the influence of the confidence coefficient on the final classification result. The generated confidence coefficients are then fed to the multi-modal module to synthesize the final diagnosis for each patient. In this way, a positive degree can better reflect the probability of MN than having a fixed threshold for a single class of each image.

### 3.3. Multi-Modal Fusion Module

Given a case, all segmented glomerular images on PASM-stained WSIs and immunofluorescence images, as well as MN confidence coefficients for glomerular images under the classification branch, could be obtained. Because WSIs are high-resolution images, detailed information about tissue morphology can be observed, while immunofluorescence images can only roughly observe the stained tissue morphology and fluorescence intensity, so WSIs and immunofluorescence images belong to two different domains. In this paper, in the diagnosis of MN, it is necessary to combine the two image domains to give a comprehensive judgment. Therefore, the multi-modal module is used to take MN probabilities on PASM-stained WSIs and immunofluorescence images into account in a complete way. Given that each patient has multiple glomeruli images in both kinds of images, the overall MN confidences $C_{P}$ and $C_{F}$ for each patient’s PASM-stained WSIs and immunofluorescence images can be calculated as follows:

(1) $$\begin{array}{c} C_{P}=\frac{1}{n}\overset{n}{\underset{i=1}{\sum }}C_{P_{i}} \end{array}$$

(2) $$\begin{array}{c} C_{F}=\frac{1}{m}\overset{m}{\underset{i=1}{\sum }}C_{F_{i}} \end{array}$$

where *n* and *m* represent the number of glomeruli in each patient’s PASM-stained WSIs and immunofluorescence images, respectively. Then, a classification model is built, such as a LASSO logistic regression model, to predict MN using a ten-fold cross-validation strategy. The probability of MN in the PASM-stained images and immunofluorescence images is aggregated into one predicted class per patient.

### 3.4. Nomogram Construction of Membranous Nephropathy

Above, the confidence coefficients $C_{P}$ and $C_{F}$ of glomeruli on PASM-stained WSIs and immunofluorescence images are added together to predict MN classification. As the classification of MN is based on two factors, a nomogram is needed to give each value of the confidence coefficient for the two types of glomeruli images. This is because each factor affects the outcome variable (the size of the regression coefficient) in a different way. The final score is calculated by adding the points from each individual’s evaluation. Through a functional transformation between the total score and the probability of MN occurrence, a predicted value for each image may be determined.

## 4. Experiments Results

This section focuses mostly on the results calculated by the proposed membranous nephropathy classification method on the kidney pathology images dataset. The recommended algorithms consist primarily of two sections, segmentation and classification networks, which are trained and tested in the same environment. As a reminder, the environment is set-up as follows: The central processing unit and graphics processing unit are comprised of an Intel(R) Xeon(R) Gold 5118 CPU at 2.30 GHz and an NVIDIA Tesla V100 PCIe GPU with 32 GB of RAM, respectively. CUDA version 10.2 is installed. The code for the entire implementation is penned in Python 3.6 using the Pytorch 1.6.0 platform and is run on a 64-bit installation of Ubuntu 18.2 LTS.

### 4.1. Datasets Preparation

A total of 1206 patients with MN and 733 patients without MN who had a renal biopsy at the Second Hospital of Shanxi Medical University were screened between 2014 and 2019. Two types of pathological image data were collected from the above cases. In total, 1206 PASM-stained WSIs and 1262 immunofluorescence images were obtained. On the one hand, renal biopsy tissues with periodic acid silver methenamine (PASM) stains were scanned to WSIs using a KF-PRO-005-EX digital slide scanner (KFbio, Ningbo, China) with a 40× objective and a 10× eyepiece at a resolution of 0.25 μm/pixel. On the other hand, fluorescence images with IgG immunoglobulins from the above patients were obtained by fluorescence microscopy with a size of 1360 × 1024. The kidney biopsy samples were all utilized for regular clinical diagnosis, and the diagnoses of all cases used in this paper were given by senior pathologists. The patient’s personal information was removed throughout the collaboration, because it would be detrimental to the patient’s privacy.

To ensure the reliability of the model, the dataset was screened. For the positive samples, the PASM-stained WSIs images with the highest-quality scanning were kept. In order to ensure that the negative samples did not interfere with the classification of MN, images of cases that were diagnosed as focal segmental glomerulosclerosis (FSGS) and minimal change disease (MCD) were selected as the normal control group. For immunofluorescence images, IgG immunofluorescence images, which are of great guideline significance for the diagnosis of MN, were selected.

Because the pathological features of membranous nephropathy are mainly manifested in the glomeruli, the glomerulus is our ROI (region of interest). The morphologically distinct glomeruli are then segmented from PASM-stained WSIs and IgG-positive immunofluorescence images of selected cases. The glomerular segmentation part is implemented by the network proposed in our previous work [30,31]. In the training phase of glomeruli segmentation, the Adam optimization algorithm is used to iterate the hyperparameter on both types of images. The batch size is set to 36, and the basic learning rate is set to 10^−3^. After 200 epoch iterations of training, testing is performed, and the weight of the glomerular segmentation network is set to the parameter with the highest Dice coefficient in testing. Finally, 8297 images of glomeruli with PASM staining from 611 patients and 1896 IgG immunofluorescence images from 1665 patients are used for confidence coefficient extraction network training, validation, and testing, as shown in Table 1. These glomeruli are all selected from sections with high staining quality and uniform thickness, and the tissues in these glomeruli are clearly visible. The confidence coefficient of the glomerulus obtained by the classification network is used to train the subsequent model fusion module. If the dataset is only divided into a training set and a test set, it is equivalent to all the data participating in the tuning of network hyperparameters, which reduces the credibility of the training data used for the model fusion module. To prevent overfitting and improve the credibility of the model, a validation set is added. During classification model training, different data augmentation techniques, such as cropping (both randomly and centered), flipping (both vertically and horizontally), and rotating, are used to make the model more reliable. It is important to note that while data augmentation techniques are used, there is no data repetition in either the training or testing datasets. In particular, glomeruli from the cases that have both the PASM-stained glomeruli and the immunofluorescence image should be considered. The glomeruli used to train and test the cross-domain aggregation model come from the test dataset of the confidence coefficient extraction network. This is performed to avoid the effects of overfitting.

### 4.2. Performance Metrics

To quantitatively evaluate the performance of the MN cross-domain classification model proposed in this paper, the following evaluation metrics are chosen: precision, recall (also known as sensitivity), and F_1_-score. These evaluation metrics play an important role in the optimization of the trained model, as they can reveal potential parts of the model that cannot be predicted correctly. The formulas of these evaluation indicators are expressed as follows:

(3) $$\begin{array}{c} Precision=\frac{TP}{TP+FP} \end{array}$$

(4) $$\begin{array}{c} Recall=\frac{TP}{TP+FN} \end{array}$$

(5) $$\begin{array}{c} F_{1}-Score=\frac{2TP}{2TP+FP+FN} \end{array}$$

where TP is True Positive, or the number of positive samples predicted to be positive; FP is false positive, or the number of positive samples that are predicted to be negative; FN is false positive, i.e., the number of negative samples predicted to be positive.

### 4.3. Performance Comparison of Different Classification Models under Different Thresholds

In order to validate the performance of classification models, Resnet [6] and Densenet [32] architectures with different layers are chosen. The classification loss function uses the cross-entropy function as follows:

(6) $$\begin{array}{c} Loss=\frac{1}{N}\sum_{i}-\left(y_{i}\log\left(P_{i}\right)+\left(1-y_{i}\right)\log\left(1-P_{i}\right)\right) \end{array}$$

where $y_{i}$ is the sample’s label: 1 indicates a positive classification and 0 indicates a negative one; $P_{i}$ is the probability that the sample i belongs to the positive classification.

After the sample data go through the classification network, the confidence level of whether the sample belongs to the category will be determined. This is a continuous number from 0 to 1 that needs to be mapped to the binary value space using a threshold.

To demonstrate the impact of the threshold, evaluation indicators such as recall, precision, and F_1_-score are listed, with thresholds of 0.3, 0.5, and 0.7, respectively, in each architecture. The results are shown in Table 2. It could be observed that the best performance of a model is different under different thresholds. Appropriate thresholds need to be found to output classification results for different application scenarios.

**Figure 4:**
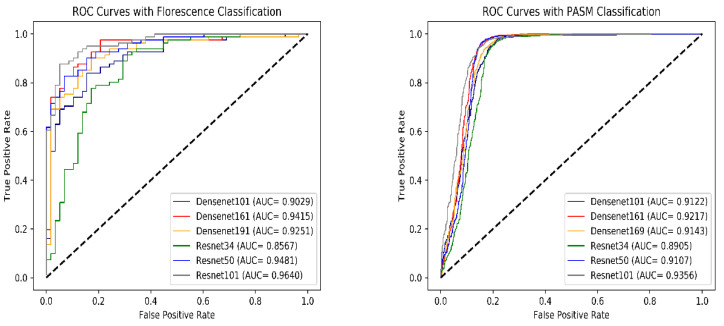
ROC curves of different models in PASM-stained WSIs (**left column**) and Immunofluorescence image (**right column**).

Figure 4 shows the ROC curves and AUC indicators for six models in PASM staining images and immunofluorescence images from the test set shown in Table 1. It is observed that Resnet101 achieves the best performance in classification models in both image domains.

### 4.4. Performance Comparison of Different Fusion Models

In the previous step, it is possible to obtain the glomerular classification model on PASM-stained WSIs and immunofluorescence images, but these two classification models can only obtain the confidence coefficient of whether a single glomerulus on a certain image belongs to MN or not. This paper introduces a multi-modal module that not only integrates the classification results of the two image domains but also the classification results of all glomerular images of the same patient. That is to say, after the fusion module, the glomerular-level classification information is converted into patient-level classification information.

Here, the aggregation module adopts five classification models based on machine learning: logistic regression, random forest, gradient boosting classifier, Xgboost [33], and lightGBM [34].

Finally, 66 patients with MN and 42 patients without MN are used to evaluate the performance of the model that aggregates the two image domains. For these cases, immunofluorescence images as well as PASM-stained WSIs images are obtained.

Then, 10-fold cross-validation is performed to find the best model and make sure it does not fit too well. The average results are shown in Table 3. The best performance in diagnosing MN is achieved (the F_1_-score is 0.9732) when the backbone is Densenet 161 and the aggregation model is logistic regression. The fusion model is compared with the best classification results obtained when only PASM-stained WSIs or immunofluorescence images are used on the same backbone. The classification results improve to a certain extent, and the improvement is shown in the sixth column of Table 3. It can also be seen in the table that, in the case of the same backbone, compared with the highest F_1_-score on a single modality without the model fusion method, the model fusion method does show a certain improvement in the MN classification performance. The aggregation models improve F_1_-scores by an average of 4.5 percent (the maximum is 5 percent).

The results of classification using images of different modalities are compared in Table 4. When only using the CNN network for MN classification of PASM-stained WSIs or immunofluorescence images, the best performing network is Resnet50, and the best F_1_-scores are 0.9276 and 0.9320, respectively. When the method proposed in this paper is used for MN classification with images of two modalities, the F_1_-score reaches 0.9732. This proves that the method proposed in this paper is effective.

Furthermore, Resnet101’s AUC indicators in PASM staining images and immunofluorescence images are both highest, whereas performance is not achieved after aggregation models. This is probably because, even if the AUC indicator weakens the impact of threshold selection, the confidence coefficient, compared with the classification result, can better reflect the cognitive level of the samples.

### 4.5. Construction of the Aggregation Nomogram

**Figure 5:**
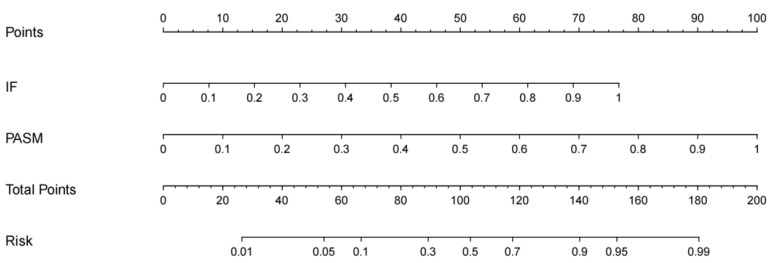
MN risk nomogram. IF and PASM denote two confidence coefficients.

The above experiments demonstrate the effectiveness of the aggregation model constructed based on the confidence coefficients obtained from PASM images and immunofluorescence image classification models for classifying MN. MN’s classification performance improves after the aggregation model. According to the state-of-the-art models in this study (logistic regression with Densenet 161), an aggregation nomogram incorporating these two coefficients is built in Figure 5. By connecting its corresponding value to the Points line in an upward-sloping line, each confidence coefficient is assigned a point value. Multiple sclerosis risk is proportional to the number of points on the Total Points line.

Through the nomogram, the scores of the confidence coefficients of the two images can be added to obtain the total score, and finally, the predicted value of whether the individual has MN is calculated through the functional conversion relationship between the total score and the probability of whether it is MN.

## 5. Conclusions

This paper proposes a classification algorithm for membranous nephropathy using a deep learning algorithm and a multi-modal fusion algorithm. PASM-stained WSIs and immunofluorescent images are combined for the classification of MN. The framework used in this paper is mainly divided into three modules: region-of-interest extraction module, confidence coefficient generation module, and multi-modal fusion. Among them, the region-of-interest extraction module identifies and segments the glomeruli on the two types of images to reduce the influence of other surrounding tissues on the subsequent confidence coefficient generation module. The confidence coefficient generation module obtains the confidence coefficients of glomeruli on the two modalities of images by training an MN classifier. Multi-modal fusion fuses the confidence coefficients of glomeruli across the two modalities through machine learning algorithms to obtain the final classification result. The results show that via logistic regression with Densenet 161, the F_1_-score of MN classification using images of two modalities achieves 0.9732 and is about 5 percentage points higher than that of images using only one modality, which proves that the research idea is feasible.

Under experimental conditions, this deep learning method can provide adjuvant assessment comparable to that of advanced pathologists. Compliance bias can occur because there are not many images of kidney pathology, light microscopy and immunofluorescence microscopy only show a small number of features, images are not spread out evenly in some categories, among others.

Although deep convolutional neural networks have shown promising results in automatically identifying kidney tissue, lesions, and related markers, the accuracy of these networks mainly depends on the size, complexity, algorithmic structure, and noise of the dataset used. Learning algorithms for detecting digital image heterogeneity such as lesions require further study.

## Figures and Tables

**Table 1:** The detailed information of image-level and patient-level datasets.

		PASM			Immunofluorescence			Fusion
		train	val	test	train	val	test	10-fold cross-validation
Patient-level	positive	194	47	66	885	209	100	66
	negatives	120	81	103	378	51	42	42
Image-level	positive	3603	914	1353	992	248	81	1353
	negatives	1534	171	722	463	42	70	70

**Table 2:** Performance comparison of different images.

Backbone	PASM Stained Images				Immunofluorescence Images			
	Threshold	Recall	Precision	F_1_-Score	Threshold	Recall	Precision	F_1_-Score
Resnet34	0.3	0.8752	0.8900	0.8825	0.3	0.6475	0.7804	0.7077
	0.5	0.8993	0.9070	0.9031	**0.5**	**0.7770**	**0.7951**	**0.7859**
	**0.7**	**0.9046**	**0.9081**	**0.9063**	0.7	0.7670	0.7898	0.7833
Resnet50	0.3	0.9200	0.9250	0.9225	0.3	0.8345	0.8711	0.8524
	0.5	0.9234	0.9265	0.9250	**0.5**	**0.9281**	**0.9360**	**0.9320**
	**0.7**	**0.9267**	**0.9285**	**0.9276**	0.7	0.8849	0.8956	0.8902
Resnet101	0.3	0.9128	0.9173	0.9150	0.3	0.6115	0.7669	0.6804
	0.5	0.9176	0.9208	0.9192	0.5	0.6835	0.7949	0.7350
	**0.7**	**0.9200**	**0.9220**	**0.9210**	**0.7**	**0.8993**	**0.8991**	**0.8992**
Densenet121	0.3	0.8564	0.8823	0.8692	0.3	0.7410	0.7926	0.7659
	0.5	0.8872	0.9034	0.8953	**0.5**	**0.8201**	**0.8204**	**0.8203**
	**0.7**	**0.9075**	**0.9142**	**0.9108**	0.7	0.7914	0.8174	0.8042
Densenet161	**0.3**	**0.9234**	**0.9278**	**0.9256**	0.3	0.8633	0.8774	0.8703
	0.5	0.9210	0.9214	0.9212	0.5	0.8705	0.8702	0.8703
	0.7	0.9051	0.9050	0.9050	**0.7**	**0.8705**	**0.8852**	**0.8778**
Densenet169	0.3	0.9012	0.9121	0.9066	0.3	0.8273	0.8511	0.8391
	**0.5**	**0.9128**	**0.9164**	**0.9146**	**0.5**	**0.8561**	**0.8561**	**0.8561**
	0.7	0.9027	0.9021	0.9024	0.7	0.8058	0.8441	0.8245

**Table 3:** Performance comparison of different fusion models.

Backbone	Fusion Model	Precision	Recall	F_1_-Score	Improvement
Resnet34	Logistic regression	0.9471	0.9390	0.9430	0.0498
	Random forest	0.9542	0.9467	0.9505	
	Gradient boosting classifier	0.9480	0.9400	0.9440	
	Xgboost	0.9582	0.9515	0.9549	
	lightGBM	**0.9597**	**0.9526**	**0.9561**	
Resnet50	Logistic regression	0.9670	0.9621	0.9646	0.0404
	Random forest	**0.9742**	**0.9708**	**0.9724**	
	Gradient boosting classifier	0.9532	0.9460	0.9496	
	Xgboost	0.9664	0.9602	0.9633	
	lightGBM	0.9640	0.9575	0.9607	
Resnet101	Logistic regression	0.9595	**0.9519**	0.9557	0.0353
	Random forest	0.9590	0.9479	0.9534	
	Gradient boosting classifier	0.9560	0.9482	0.9521	
	Xgboost	0.9537	0.9420	0.9478	
	lightGBM	**0.9608**	0.9518	**0.9563**	
Densenet121	Logistic regression	0.9560	0.9458	0.9509	0.0472
	Random forest	0.9593	0.9524	0.9559	
	Gradient boosting classifier	0.9390	0.9245	0.9317	
	Xgboost	0.9611	0.9517	0.9564	
	lightGBM	**0.9619**	**0.9542**	**0.9580**	
Densenet161	Logistic regression	**0.9748**	**0.9716**	**0.9732**	0.0476
	Random forest	0.9531	0.9469	0.9732	
	Gradient boosting classifier	0.9359	0.9260	0.9301	
	Xgboost	0.9504	0.9414	0.9458	
	lightGBM	0.9571	0.9545	0.9596	
Densenet169	Logistic regression	**0.9670**	**0.9621**	**0.9646**	0.0500
	Random forest	0.9603	0.9525	0.9563	
	Gradient boosting classifier	0.9408	0.9328	0.9368	
	Xgboost	0.9542	0.9444	0.9492	
	lightGBM	0.9658	0.9603	0.9630	

**Table 4:** Performance comparison of different image models.

	Image Modal	Precision	Recall	F_1_-Score
Resnet 50	PASM stained WSIs	0.9267	0.9285	0.9276
Resnet 50	Immunofluorescence images	0.9281	0.9360	0.9320
Densenet161 + Logistic regression	PASM stained WSIs + Immunofluorescence images	0.9748	0.9716	0.9732

## Data Availability

Not applicable.
